# Pectoralis Major Rupture: A Case Report of a 34-Year-Old Rugby Player

**DOI:** 10.7759/cureus.73506

**Published:** 2024-11-12

**Authors:** Nawara Aljasim, Fahad Alkhalifa

**Affiliations:** 1 Internal Medicine, King Hamad University Hospital, Busaiteen, BHR; 2 Orthopedic Surgery, Bahrain Defence Force Hospital, Riffa, BHR

**Keywords:** ortho surgery, pectoralis major rupture, shoulder injuries, sport injury, trauma

## Abstract

Pectoralis major injuries are not commonly seen; this type of injury occurs mainly due to weightlifting and bench pressing. The majority of cases occur in men between the ages of 20 to 40 years. We present a case of a previously healthy 34-year-old male who presented to the emergency department after sustaining an injury to the left shoulder and loin while playing rugby. Physical examination revealed a swelling over the deltopectoral region, with a large hematoma noted over the left loin. The patient had mild pain over the left shoulder, chest, and loin. He then went to an orthopedic surgeon seven days later, and an ultrasound was performed, revealing a partial pectoralis muscle rupture. The patient underwent surgical repair on the same day, and an intraoperative examination showed a complete pectoralis major tear, which was successfully repaired.

## Introduction

Pectoralis major muscle rupture is a rare injury with increasing incidence in the last decades; it most commonly occurs in bodybuilders and athletes. The majority of cases diagnosed as pectoralis major rupture are between the ages of 20 to 40 years, being mainly male predominant injury [1].

The pectoralis major muscle is the largest muscle found in the anterior chest wall. It is a fan-shaped muscle containing two heads and lies under the breast tissue. A clavicular head originates from the medial half of the clavicle anteriorly. The sternocostal head originates from the manubrium, the body of the sternum, the superior six costal cartilages, and the superior part of the external oblique muscle aponeurosis. These fibers unite and insert into the humerus, lateral to the bicipital groove. The primary function of the pectoralis muscle is internal rotation and adduction of the humerus, in addition to humeral flexion, when the arm is extended posterior to the coronal plane. The clavicular head is innervated by the lateral pectoral nerve, while the sternocostal head is innervated by the medial pectoral nerve [2].

## Case presentation

A previously healthy 34-year-old male presented to the emergency department on the 13th of January 2024 after sustaining an injury to the left shoulder and loin while playing rugby. The mechanism of injury occurred while the patient was in a scrum position, which involved eight players from each team binding together and interlocking in three rows pushing against each other. The patient's arms were placed around his teammates while pushing against the other team, his arm was forcefully abducted and externally rotated, and a pop was heard associated with a tearing sensation felt on the left side of the chest and shoulder. On general examination, the patient was vitally stable, alert, and oriented. A swelling was noted over the deltopectoral region, and a large hematoma was seen over the left loin. The patient had mild pain over the left shoulder, chest, and loin. In the emergency department, the patient was assessed for any life-threatening injuries, where an extended focused assessment with sonography in trauma (E-FAST) scan was performed, revealing a hematoma in the left loin measuring 2.6x2.3 cm. The patient was deemed stable for discharge from the emergency department and was referred to the orthopedic department for further evaluation. Meanwhile, he was given analgesia, and his left arm was immobilized with a sling and cold compression, as advised by the emergency doctor.

**Figure 1:**
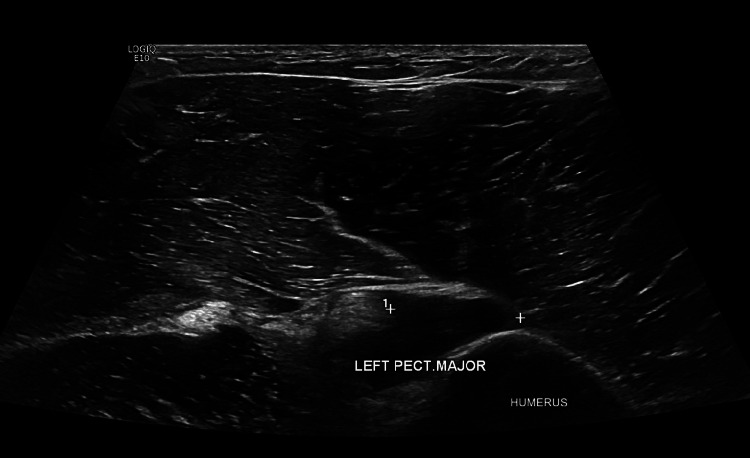
Ultrasound scan of the left shoulder Partial dislodgment of the distal fibers at the musculotendinous aspect of the pectoralis muscle at the humeral insertion with a partial gap of around 15 mm from the humeral attachment was observed.

On the 21st of January 2024, the patient underwent an ultrasound scan of the left shoulder ordered by the orthopedic consultant, which showed partial dislodgment of the distal fibers at the musculotendinous aspect of the pectoralis muscle at the humeral insertion with a partial gap measuring 15 mm from the humeral attachment highly suggestive of partial pectoralis rupture. Moreover, moderate synovitis of the long head of the biceps was observed. A non-homogenous echo pattern measuring 35x19 mm suggestive of grade II-III sprain of the pectoralis muscle was noted as per the ultrasound report (Figure 1).

**Figure 2:**
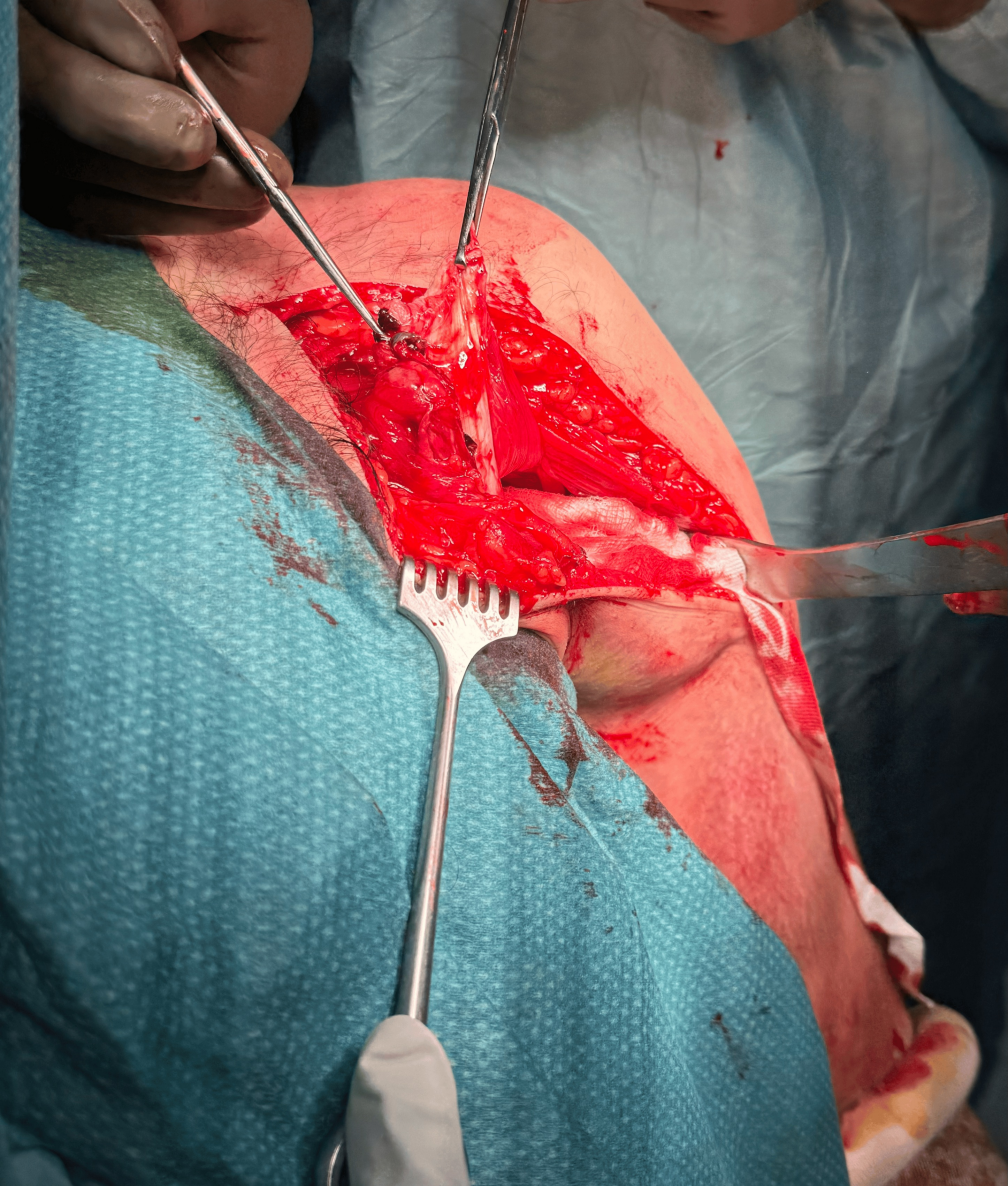
Intraoperative Image A complete left-sided pectoralis major tear was noted.

After an initial diagnosis with the ultrasound, the patient was offered surgical repair, which he accepted. The surgery was scheduled on the same day. Intraoperative examination revealed a complete pectoralis major tear with a small tendon remaining at the attachment site (Figure 2). This injury was classified as type III C according to the Tietjen classification. During the procedure, tendon repair was done by stitches, which were taken through the pectoralis major tendon, and two holes were drilled lateral to the bicipital groove. The stitches were then passed through two endobuttons, and these endobuttons were inserted into the drilled holes. The muscle ends were approximated, and knots were placed to fix the endobuttons in place. Postoperatively, the patient was vitally and clinically stable; therefore, he was discharged in good and stable condition on the same day and was given a follow-up appointment at the clinic. He was followed up at the orthopedic outpatient department one week postoperatively. The patient was doing well, and the wound was healing properly. He then had regular monthly follow-ups for three months and was referred for physiotherapy. 

## Discussion

Pectoralis major ruptures are rare and mainly occur in young, active males. Patients with such injuries present with varying symptoms depending upon the severity and classification of the injury. The presenting complaint in such patients might be described as a feeling of something being torn or hearing a pop, along with the tearing sensation felt in the anterior chest wall. In addition, a patient might complain of pain at the tear site with a limited range of motion, which could be associated with swelling, tenderness, and ecchymosis. The physical examination may reveal an asymmetrical chest, where the nipple on the side of the injury would lie inferior compared to the nipple of the normal uninjured side. Some patients might have abnormal bulging seen in the anterior chest, indicating that the pectoralis muscle had retracted medially. Asking the patients to raise their hands and place them on their hips can lead to the separation of the humerus from the torn muscle, leading to the webbed appearance in the anterior axilla [1].

The mechanism of rupture is either due to direct or indirect trauma to the muscle, as the muscle is put under extreme tension, causing it to rupture. The case presented was a result of a rugby game, which is seen in a minority of previously documented reports. The vast majority of cases were due to weightlifting injuries, accounting for 50% of cases found in the literature. Moreover, the initial evaluation in terms of diagnosing patients with a ruptured pectoralis major muscle starts by taking a thorough history and physical examination, as high clinical suspicion is needed to evaluate the patients further and manage them effectively. Imaging modalities such as radiography are often done as part of the initial assessment to diagnose any acute bony avulsion or fractures, although they are of limited use in diagnosing muscle ruptures [3]. The main imaging modality used to confirm the diagnosis is magnetic resonance imaging (MRI) of the chest. It would identify the precise location of the tear, in addition to assessing for hematomas, tendon retractions, and hemorrhages, which would be helpful for the surgical procedure. MRI is not operator-dependent, and it is a common imaging modality used in most patients with injuries to the pectoralis muscle, as it enables better differentiation of the injury. ElMaraghy et al. briefly stated that MRI has been useful in identifying full-thickness ruptures. Although, it is less reliable in diagnosing partial thickness tears. Furthermore, multiple resources mention that it is only at the time of surgical repair that the true morphology of the rupture is characterized [4].

Moreover, it has been proven that ultrasound is an equally effective modality in diagnosing pectoral major ruptures. Ultrasound use in pectoralis major injuries was first described by Pavlik and colleagues, who explained its use in some cases to avoid surgical delay as it has been proven to be an inexpensive and efficient way of identifying and diagnosing ruptures. It would be identified by seeing an uneven echogenicity and thinning of the muscle on the affected side [5]. The role of ultrasounds in differentiating between partial or full-thickness tears was not fully investigated. Although, according to Weaver et al., ultrasounds can be used to identify disruptions of the musculotendinous junction, retraction of the muscle fibers, and surrounding hemorrhage [6]. According to Lee et al., ultrasound scans are readily available and facilitate higher spatial resolution than MRI, but an experienced sonographer is required [7].

In the presented case, the diagnosis was confirmed by an ultrasound, and the patient was taken to the operative room on the same day of diagnosis, avoiding surgical delay. Further confirmation of a complete rupture was made during the intraoperative examination. When reviewing the literature, a case report of a 28-year-old male weightlifter presenting to the emergency department with the complaint of acute left shoulder pain while weightlifting and further evaluation concluded the diagnosis of a ruptured pectoralis major muscle done with a quick point of care ultrasound imaging at the emergency department. This ultrasound imaging result allowed prompt diagnosis and further management, including the appropriate referral to the orthopedic department [8].

The classification criteria, first proposed by Tietjen in 1980, classified the location and severity of the injury to the muscle. Tietjen denoted that injuries classified as type I consist of muscle contusions or sprain, type II are partial tears and type III are complete tears. Type III is further subdivided according to the location as follows: A - muscle origin, B - muscle belly, C - musculotendinous junction, and D - tendinous insertion. Moreover, a subclassification has been proposed by Bak et al. for bony avulsion and muscle-tendon substance rupture [9]. In 2012, ElMaraghy and Devereaux analyzed 365 published cases of pectoralis muscle injury and proposed a contemporary and comprehensive classification system. It incorporates the timing of the injury, meaning if the injury was acute or chronic and anything beyond six weeks was considered a chronic injury. The second component was the location of the tear, which would affect the management and repair technique. It was simplified into three locations: first is the injury at the muscle origin or belly, the second is at or between the musculotendinous junction and tendinous insertion, and the third is bony avulsion at the humerus. Finally, the extent of the tear, if it involved the anterior to posterior thickness, and whether it was a complete or incomplete tear [4].

Managing a patient diagnosed with a ruptured pectoralis muscle includes both a conservative approach and surgical repair, as per the literature. Conservative management is usually reserved for people who refuse to undergo surgical repair, older patients, or those who are deemed unfit for surgery. Conservative management entails applying a sling to the affected limb, cryotherapy to control the swelling, and pain control with analgesics in addition to physiotherapy. There is minimal literature regarding the optimal time for surgical intervention. Although, it has been mentioned that acute surgical interventions are easier, leading to better outcomes. Chronic surgical intervention may lead to the appearance of adhesion between the ruptured muscle and the chest wall, leading to a complicated surgical procedure [9]. Furthermore, multiple surgical techniques have been reported, such as trans-osseous fixation, suture fixation, anchor fixation, and cortical button fixation. In addition, autografts or allografts are used to repair chronic ruptures [10]. According to Antosh et al., repairs done within the six-week window had the best overall results and recovery optimization [11]. A meta-analysis of 112 cases of pectoralis major rupture by Bak et al. highlighted that patients who opt for surgical repair have a much higher chance of returning to preinjury function level and activities than patients who are treated conservatively [12]. Moreover, Hanna et al. argued that patients who had undergone surgical repair regained 97% of their strength compared to 56% in patients treated conservatively [13].

Our patient was young, active, and athletic; therefore, he was managed with prompt surgical repair, which was within nine days of his injury. The pectoralis muscle was repaired with the use of endobuttons and sutures. The patient was first given a follow-up appointment at the orthopedic outpatient department one week postoperatively. No postoperative complications were noted; he then had regular monthly follow-ups for three months, during which he was referred for physiotherapy. The patient now has a normal range of motion and muscle power.

## Conclusions

In conclusion, pectoralis major ruptures are uncommon, and few cases have been reported in the literature. It mainly occurs in young and athletic males. Patients might present with pain or weakness at the site of injury; a diagnosis can either be made by an MRI or an ultrasound scan. Surgical repair has favorable outcomes, and conservative management is an option reserved for people who refuse surgical repair or the elderly population.
